# Optimization of Fermentation Conditions for the Production of 2,3,5-Trimethylpyrazine by Recombinant *Bacillus licheniformis*

**DOI:** 10.3390/microorganisms13071477

**Published:** 2025-06-25

**Authors:** Xun Liu, Hongyi Gu, Handong Wang, Zhen Tang, Shuanglian Chen, Han Li, Wenli Quan

**Affiliations:** 1 School of Food and Liquor Engineering, Sichuan University of Science & Engineering, Yibin 644000, China; xunliu0123@hotmail.com (X.L.);; 2 Brewing Science and Technology Key Laboratory of Sichuan Province, Sichuan University of Science & Engineering, Yibin 644000, China; 3 Liquor Making Biotechnology and Intelligent Manufacturing of Key Laboratory of China National Light Industry, Yibin 644000, China; 4 Qinghai Provincial Key Laboratory of Crop Molecular Breeding, Northwest Institute of Plateau Biology, Chinese Academy of Sciences, Xining 810008, China

**Keywords:** 2,3,5-trimethylpyrazine, *Bacillus licheniformis*, *BlTDH* gene, recombinant strains, fermentation optimization

## Abstract

2,3,5-Trimethylpyrazine (TMP) is an alkyl pyrazine with broad application prospects in the fields of food additives and medicine. L-threonine-3-dehydrogenase (TDH) is a key enzyme in the biosynthesis pathway of TMP. To explore the efficient and environmentally friendly production method of TMP, we constructed recombinant strains overexpressing the *BlTDH* gene and its mutant *BlTDH* (N157A) using *Bacillus licheniformis* YC7. The TMP yield of recombinant strains with pHT01-*BlTDH* (N157A) reached 15.35 ± 1.51 mg/L, which was significantly higher than that of strains with pHT01-*BlTDH* (9.86 ± 1.24 mg/L) and strains with vector pHT01 (2.35 ± 0.58 mg/L). To further increase the TMP yield of strain YC7/pHT01-*BlTDH* (N157A), the fermentation process was optimized by single-factor experiments, and the response surface test was conducted using the Box–Behnken design. The results revealed that the substrate ratio, IPTG concentration and fermentation time had significant effects on the yield of TMP, and the degree of influence was substrate ratio > fermentation time > IPTG concentration. The optimization results of response surface indicated that the optimal fermentation conditions were as follows: substrate ratio of 1:2, IPTG concentration of 1.0 mM, and fermentation time of 4 d. Under these conditions, the TMP yield reached 44.52 ± 0.21 mg/L, which was around 0.005 mg/L different from the predicted value (45.515 mg/L), and increased by 29.17 mg/L compared with the initial condition. The optimization of fermentation conditions significantly increased the yield of TMP produced by recombinant strains, which provided a theoretical basis and strain resources for industrial production of TMP.

## 1. Introduction

**Figure 1:**
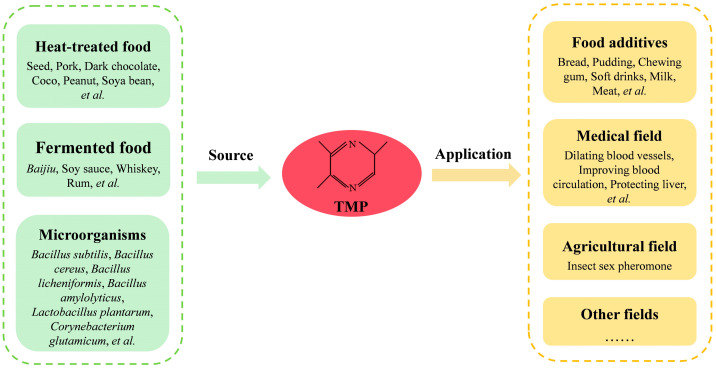
The source and application of TMP.

Alkyl pyrazines are important flavor substances in food. Although their sensory thresholds are low, they have a significant impact on the flavor and quality of food [1]. 2,3,5-trimethylpyrazine (TMP) is a multifunctional alkyl pyrazine compound. It can not only be used in the food flavoring industry but also exhibits various physiological activities in the medical field, such as dilating blood vessels, improving blood circulation and protecting the liver [2,3] (Figure 1). TMP is commonly found in heat-treated seeds [4], roasted pork [5], dark chocolate [6], cocoa [7], roasted peanuts [8] and other foods. It is also an important flavor component in traditional fermented products such as soy sauce [9], whisky [10], rum [10] and *Baijiu* [11]. In traditional Chinese *Baijiu*, the content of TMP ranges from 17.39 to 1428.41 μg/kg [11]. Furthermore, TMP can serve as a sex pheromone for male fruit flies [12]. There are two main types of synthesis routes of TMP: chemical synthesis and microbial synthesis. Chemical synthesis requires a high temperature and is difficult to control precisely, while microbial synthesis, due to its mild conditions, environmental friendliness and high product specificity, has become the main method for TMP production [13].

**Figure 2:**
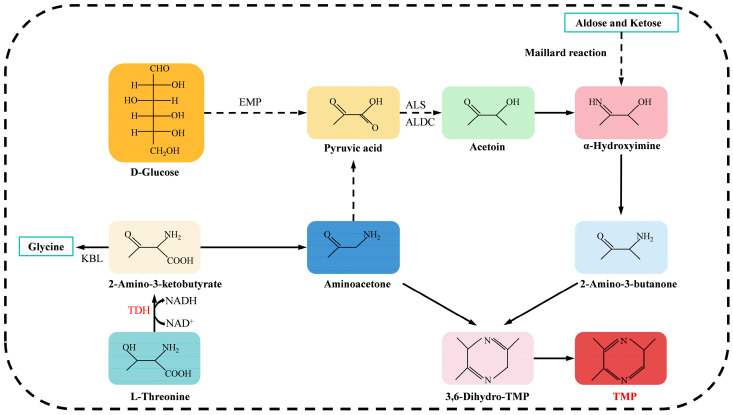
The biosynthesis pathway of TMP.

Currently, a variety of microorganisms have been confirmed to have the ability to synthesize TMP, including *Bacillus subtil*is [14], *B. cereus* [15], *B. licheniformis* [16], *B. amyloliquefaciens* [16], *Lactobacillus plantarum* [17], *Corynebacterium glutamicum* [18], etc. (Figure 1). Zhang et al. [19] clarified the microbial synthesis mechanism of TMP, and this process mainly involves two metabolic pathways (Figure 2). On the one hand, D-glucose generates pyruvic acid through glycolysis, and then is converted to acetoin under the catalytic action of α-acetyllactate synthase (ALS) and α-acetyllactate decarboxylase (ALDC), ultimately generating 2-amino-3-butanone. On the other hand, L-threonine is oxidized to 2-amino-3-ketobutyric acid under the catalysis of L-threonine dehydrogenase (TDH), and the latter forms amino-acetone through a spontaneous decarboxylation reaction. The two precursors, derived from D-glucose and L-threonine, respectively, eventually synthesize TMP through spontaneous condensation reactions.

Although the production of TMP through microbial fermentation has significant environmental advantages, the current production efficiency is still difficult to meet industrial demands [11]. To address the low yield of TMP, researchers have adopted a variety of optimization strategies so far. As an effective tool to optimize microbial fermentation parameters, the response surface method has been widely applied in the optimization of metabolite production process [20,21]. By establishing mathematical models, regression analysis and analysis of variance, the optimal parameter combination of the fermentation process can be determined. Among them, the Box–Behnken design is a commonly used statistical model in the response surface method [22,23]. Liu et al. [16] isolated and screened a strain of *B. amyloliquefaciens* with a strong ability to produce TMP from *Daqu*. Through the optimization of fermentation conditions, the TMP content was successfully increased from 0.071 mg/g to 0.446 mg/g, significantly improving the production efficiency of TMP. With the in-depth study of TMP biosynthesis pathway, Zhang et al. [19] found that the overexpression of the *BsTDH* gene in *B. subtilis* 168 can effectively enhance the yield of TMP.

Previously, we screened out the *Bacillus licheniformis* YC7 strain capable of producing TMP, and the *BlTDH* gene was cloned from *Bacillus licheniformis* YC7 strain. Five BlTDH mutants were constructed by site-directed mutagenesis, in which the BlTDH (N157A) mutant was selected based on systematic screening. Specifically, the BlTDH (N157A) mutant had significantly enhanced enzyme activity compared to wild-type BlTDH. In this study, we constructed a recombinant strain with an overexpression of *BlTDH* and *BlTDH* (N157A). The fermentation conditions of TMP production were systematically optimized by using the single-factor experiments combined with the response surface method. The effects of substrate ratio (ratio of D-glucose to L-threonine), IPTG concentration and fermentation time on the yield of TMP were investigated. This study will establish an efficient TMP biosynthesis process and provide a theoretical basis and available strain resources for the industrial production of TMP.

## 2. Materials and Methods

### 2.1. Materials and Culture Medium

#### 2.1.1. Strains and Plasmids

The *BlTDH* gene was cloned from the genomic DNA of *B. licheniformis* YC7. The strains and plasmids used in this study were shown in Table 1.

#### 2.1.2. Culture Medium

The LB liquid medium was prepared with 10 g/L peptone, 5 g/L yeast extract, and 10 g/L NaCl, with a pH of 7.0, and sterilized at 121 °C for 20 min. In addition, 20 g/L agar powder was included in the solid medium [24]. On the basis of LB medium, the fermentation medium contained 5 g/L L-threonine, 10 g/L D-glucose and 3 g/L diammonium hydrogen phosphate, with pH 7.0 [19].

### 2.2. Construction of Recombinant Strains

The laboratory-preserved recombinant plasmids, pET-28a-*BlTDH* and pET-28a-*BlTDH* (N157A), were used as templates for PCR amplification of target genes. Specific primers containing the digestion sites of *Bam*H I and *Xho* I were designed using SnapGene 6.0.2 software. Primer sequences: forward: 5′-CGCGGATCCATCTTGGACGGAATGAAAGCGCT-3′, reverse: 5′-CCGCTCGAGCTACGGTATCAGTACGACTTTGC-3′. The PCR products of *BlTDH* and *BlTDH* (N157A) were purified and then connected to the pHT01 vector. After verification by Sanger sequencing, the recombinant plasmids, pHT01-*BlTDH* and pHT01-*BlTDH* (N157A), were successfully obtained. Then, these recombinant plasmids were transformed into *B. licheniformis* YC7 competent cells according to the method reported by Spizizen [25]. To ensure a similar transformation efficiency, we used identical competent cells, equal amounts of recombinant plasmids, and the same transformation method. Additionally, after the transformation, a PCR test was conducted for verification.

### 2.3. Determination of TMP Yield of Recombinant Strains

The recombinant strains with pHT01, pHT01-*BlTDH,* or pHT01-*BlTDH* (N157A) vectors were inoculated into LB liquid medium with 100 μg/mL ampicillin. After overnight incubation at 37 °C, a 5 mL inoculum was transferred to 100 mL of fermentation medium. When the OD_600_ value reached 0.6–0.8, IPTG was added to make its final concentration 1.0 mM. After continuous culture at 25 °C and 180 rpm for 48 h, the content of TMP was detected by gas chromatography–mass spectrometry (GC-MS). To enable accurate comparison of TMP yields, all culture samples were normalized to OD_600_ = 1.0 prior to TMP extraction and GC-MS analysis.

The analytical conditions for GC-MS were as follows: the specifications of DB-WAX capillary column were 60 m × 250 μm, 0.25 μm. The inlet temperature was 230 °C. The programmed heating process was set as follows: the initial temperature was 40 °C, stabilized for 3 min, increased to 120 °C with a rate of 5 °C/min, raised to 230 °C at a rate of 7 °C/min, and then held for 10 min. MS parameters: ion source temperature and interface temperature were 230 °C, with a scanning range of 20–500 u and electron energy of 70 eV [16].

### 2.4. Single-Factor Experimental Design

*B. licheniformis* YC7/pHT01-*BlTDH* (N157A) was selected to study the effects of substrate ratio, IPTG concentration and fermentation time on TMP yield according to the method of single-factor analysis. Using the controlled variable method, the following parameters were tested: substrate ratios of 0:1, 1:2, 1:1, 2:1 and 1:0 (with fixed IPTG concentration of 1.0 mM and fermentation time of 2 d). The IPTG concentration was, respectively set as 0.2 mM, 0.6 mM, 1.0 mM, 1.4 mM and 1.8 mM (with fixed substrate ratio of 2:1 and fermentation time of 2 d). The fermentation time was set as 1 d, 2 d, 3 d, 4 d, and 5 d, respectively (with fixed substrate ratio of 2:1 and IPTG concentration of 1.0 mM).

### 2.5. Box–Behnken Design

The method of Box–Behnken design was employed to optimize the fermentation conditions of TMP production based on the three-factor and three-level experimental scheme [26]. According to the single-factor analysis, the optimal parameter ranges of the three factors used in the Box–Behnken design were selected and listed in Table 2. The experimental design consisted of 17 groups of independent experiments (including 5 center points). A prediction model was established through multiple regression analysis to obtain the regression equation. The three-dimensional response surface diagram was drawn using the Design-Expert 13.0 software to visualize the interaction between the various factors.

## 3. Results

### 3.1. Construction of BlTDH and BlTDH (N157A) Overexpression Strains

**Figure 3:**
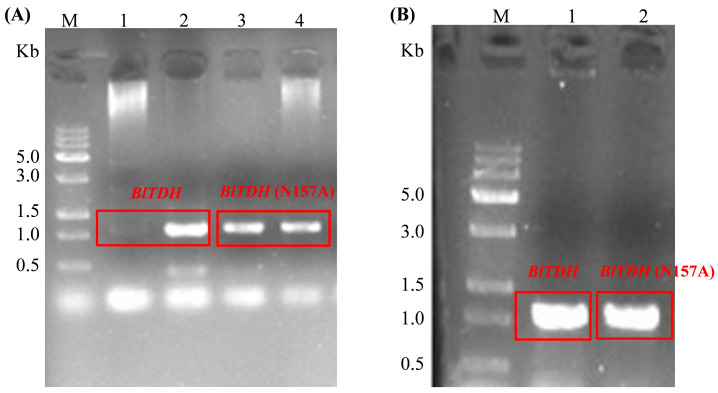
Gene cloning (**A**) and verification of recombinant strains by bacterial liquid PCR (**B**). (**A**) M: DNA marker; 1–2: Gene *BlTDH*; 3–4: Gene *BlTDH* (N157A); (**B**) M: DNA marker; 1: Gene *BlTDH*; 2: Gene *BlTDH* (N157A).

PCR amplification was performed using recombinant plasmids, pET-28a-*BlTDH* and pET-28a-*BlTDH* (N157A) (constructed in our laboratory), as DNA templates. Through agarose gel electrophoresis, it was found that the target fragments of *BlTDH* and *BlTDH* (N157A) were displayed near 1.0 Kb, which was consistent with the expected sizes (Figure 3A). After constructing the recombinant expression plasmids, pHT01-*BlTDH* and pHT01-*BlTDH* (N157A), Sanger sequencing was conducted to verify that the recombinant plasmids were successfully constructed. The result of sequencing analysis suggested that the gene sequences of *BlTDH* and *BlTDH* (N157A) were complete and correct, with a length of 1044 bp (data not show), indicating that the recombinant plasmids pHT01-*BlTDH* and pHT01-*BlTDH* (N157A) were successfully constructed.

The obtained overexpression plasmids pHT01-*BlTDH* and pHT01-*BlTDH* (N157A) were transferred into *B. licheniformis* YC7 competent cells. The positive transformation was selected and verified by bacterial liquid PCR using the upstream universal primer of the pHT01 vector and the downstream primer of *BlTDH* (Figure 3B). The size of PCR products was consistent with the theoretical expectation, indicating the successful construction of *BlTDH* and *BlTDH* (N157A) overexpression strains.

### 3.2. Fermentation Verification of BlTDH and BlTDH (N157A) Overexpression Strains

**Figure 4:**
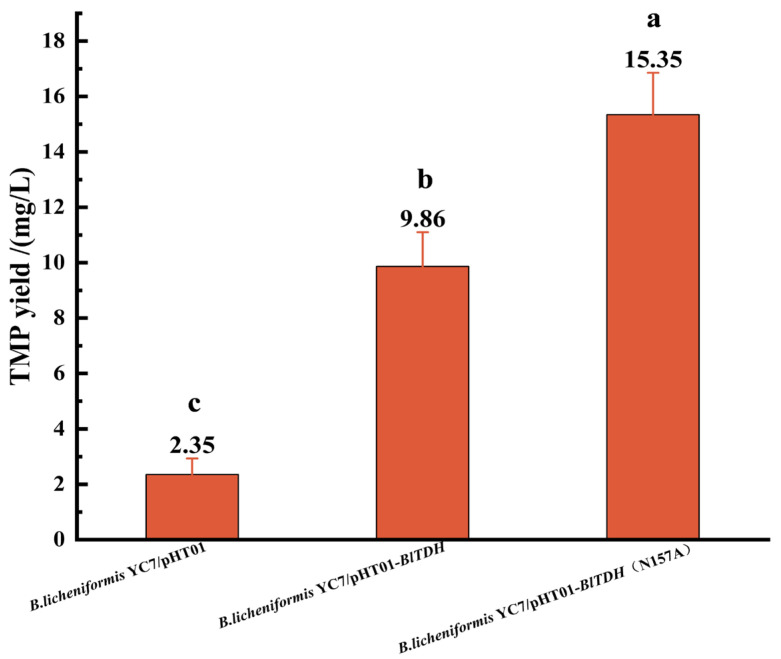
The TMP yield of different recombinant strains. The vertical bars with different lower-case letters are significantly different from each other at *p* < 0.05 (one-way ANOVA).

The constructed strains, *B. licheniformis* YC7/pHT01 (as control), *B. licheniformis* YC7/ pHT01-*BlTDH* and *B. licheniformis* YC7/pHT01-*BlTDH* (N157A), were, respectively inoculated into the fermentation medium for cultivation to produce TMP. The results showed that the TMP yield of the overexpression strains with pHT01-*BlTDH* and pHT01-*BlTDH* (N157A) were 9.86 ± 1.24 mg/L and 15.35 ± 1.51 mg/L, respectively. However, the yield of the control was only 2.35 ± 0.58 mg/L, which was obviously lower than those of the overexpression strains (Figure 4). This result demonstrated that the overexpression of *BlTDH* gene could significantly enhance the TMP synthesis ability of *B. licheniformis* YC7 strains. Meanwhile, the BlTDH (N157A) mutant further promoted the synthesis of TMP.

### 3.3. Single-Factor Analysis

#### 3.3.1. Effect of Substrate Ratio on TMP Production

To explore the influence of different factors on the yield of TMP, the recombinant strain overexpressing *BlTDH* (N157A) with the highest TMP yield under the initial fermentation condition was taken as the experimental object.

**Figure 5:**
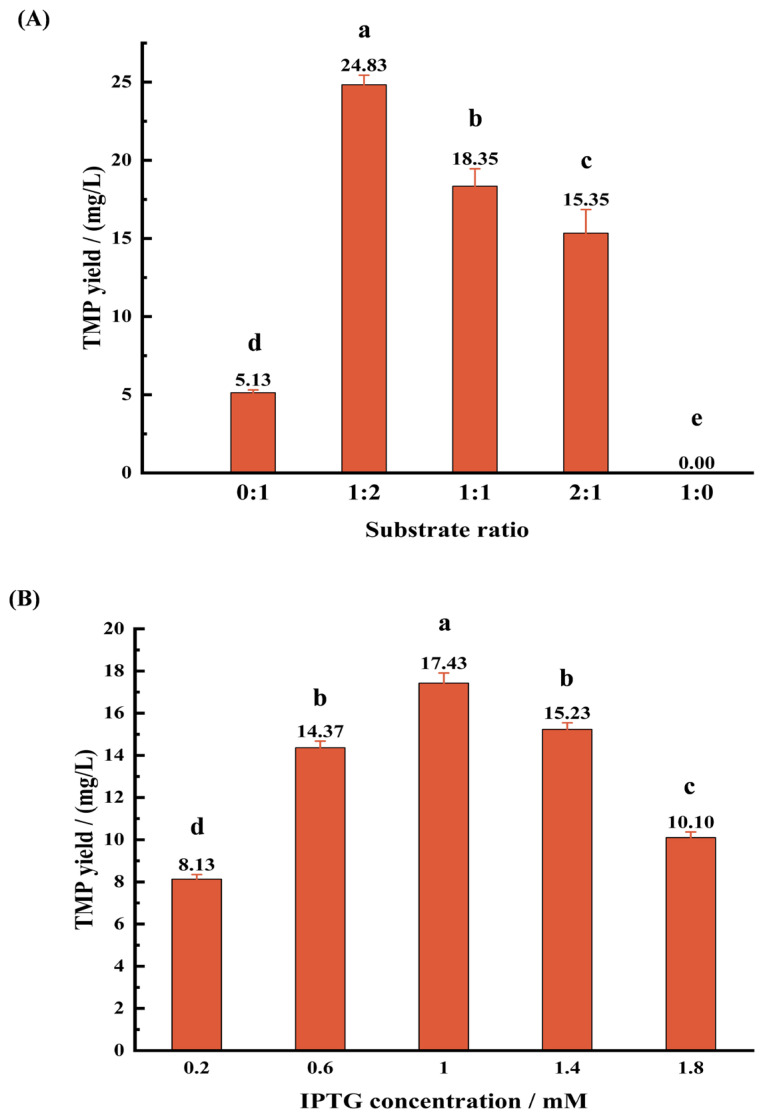

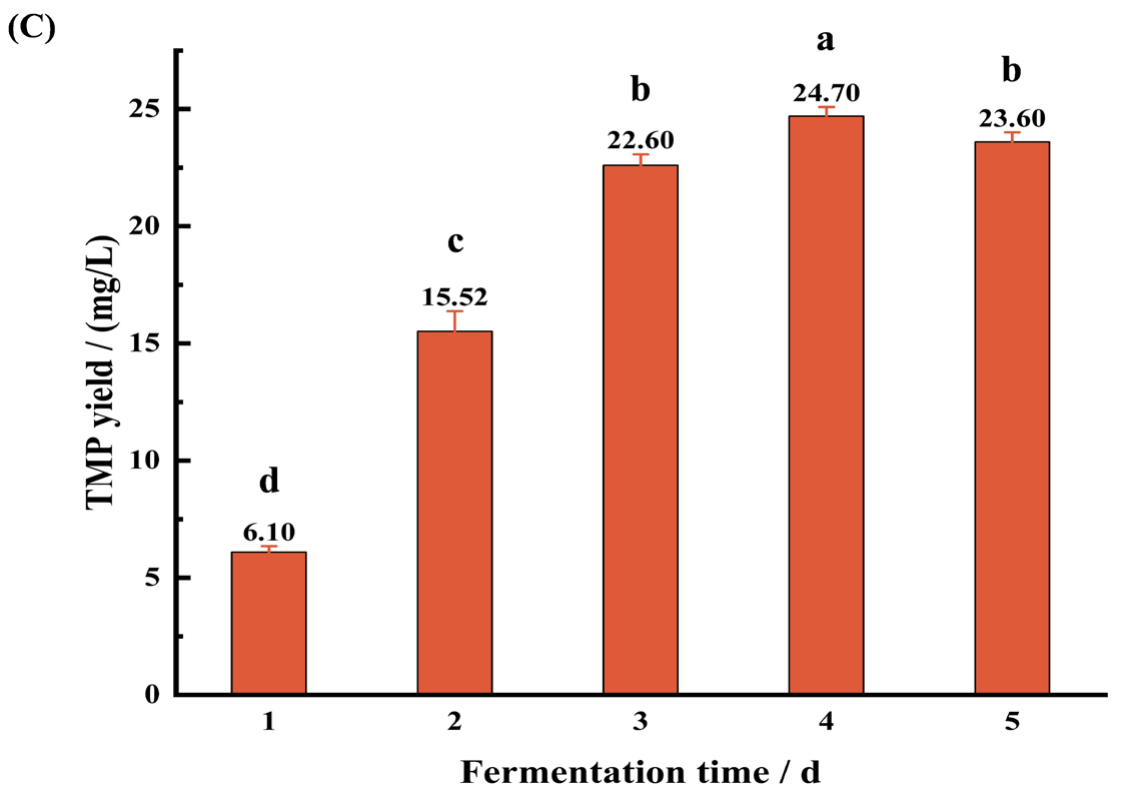
Effects of different factors on TMP yield. (**A**) substrate ratio; (**B**) IPTG concentration; (**C**) fermentation time. The vertical bars with different lower-case letters are significantly different from each other at *p* < 0.05 (one-way ANOVA).

Firstly, the effect of different substrate ratio (D-glucose:L-threonine) on TMP yield was investigated under the fixed condition of IPTG concentration (1.0 mM) and fermentation time (2 d). With the change in substrate ratio, the output of TMP first increased and then decreased. When the substrate ratio was 1:2, the TMP yield reached the maximum value, which was 24.83 ± 0.61 mg/L (Figure 5A). Therefore, a ratio of D-glucose to L-threonine of 1:2 was selected as the optimal substrate ratio for the production of TMP.

#### 3.3.2. Effect of IPTG Concentration on TMP Production

IPTG can induce the expression of *BlTDH* gene, and different concentrations of IPTG have different effects on the expression of *BlTDH*. Therefore, we studied the influence of different IPTG concentrations on the yield of TMP under the fixed condition of substrate ratio (2:1) and fermentation time (2 d). With the increase in IPTG concentration, the TMP yield presented a trend of rising first and then declining. When the concentration of IPTG was 1.0 mM, the maximum yield of TMP was 17.43 ± 0.48 mg/L (Figure 5B). Therefore, 1.0 mM IPTG was considered as the optimal concentration for the TMP production by recombinant strains.

#### 3.3.3. Effect of Fermentation Time on TMP Production

The fermentation time has a significant impact on the accumulation of microbial metabolites. Hence, the effect of fermentation time on the TMP yield was studied under the fixed condition of substrate ratio (2:1) and IPTG concentration (1.0 mM). The production of TMP gradually increased with the extension of the fermentation time, except on the fifth day. Apparently, the TMP yield reached the maximum value of 24.70 ± 0.39 mg/L at 4 d of fermentation (Figure 5C), which was selected as the optimal fermentation time for TMP biosynthesis.

### 3.4. Response Surface Analysis

Based on the results of single-factor experiments, the Box–Behnken design was performed to optimize the fermentation conditions for TMP production. The experimental design consisted of 17 treatment groups, including 12 factor combination groups and 5 center-point replicates (Table 3). As shown in Table 3, the TMP yield of 17 groups varied within the range of 13.52–44.99 mg/L, indicating that these factors and their combinations had a significant impact on the production of TMP.

Multiple regression fitting analysis was conducted on the data in Table 3 using the Design-Expert 13.0 software. The regression equation of the response surface was obtained as follows:

y = 44.42 + 1.38A + 0.6775B + 0.7888C + 0.6025AB − 3.38AC − 3.95BC − 16.47A^2^ − 12.57B^2^ − 1.76C^2^

According to the *p*-value, the order of influence of single-factor analysis on TMP production was B (0.0426) > C (0.0237) > A (0.0015), namely IPTG concentration > fermentation time > substrate ratio (Table 4). The *p*-value of the established regression model was less than 0.0001, suggesting that the model was extremely significant. The *p*-value of the fitting failure of the regression model was 0.4266, which was greater than 0.05, indicating that the pure error was not significant. In the regression model, the primary items B and C were significant with *p* < 0.05; while, the primary item A, the interaction terms AC and BC, and the secondary terms A2, B2 and C2 were highly significant with *p* < 0.01, demonstrating that these factors and their interaction had an important influence on the TMP yield. The coefficient of determination R^2^ was 0.9980, implying that the regression model fitted well with the experimental data. The adjusted coefficient of determination R^2^_Adj_ was 0.9954, which further confirmed that each factor in the regression model had a significant impact on the TMP yield. The difference between R^2^_Adj_ and R^2^_pred_ was less than 0.2, indicating that this regression model was effective, could fully explain the technological process, and could be used to analyze the influence of various factors on the TMP yield.

### 3.5. Response Surface Interaction

**Figure 6:**
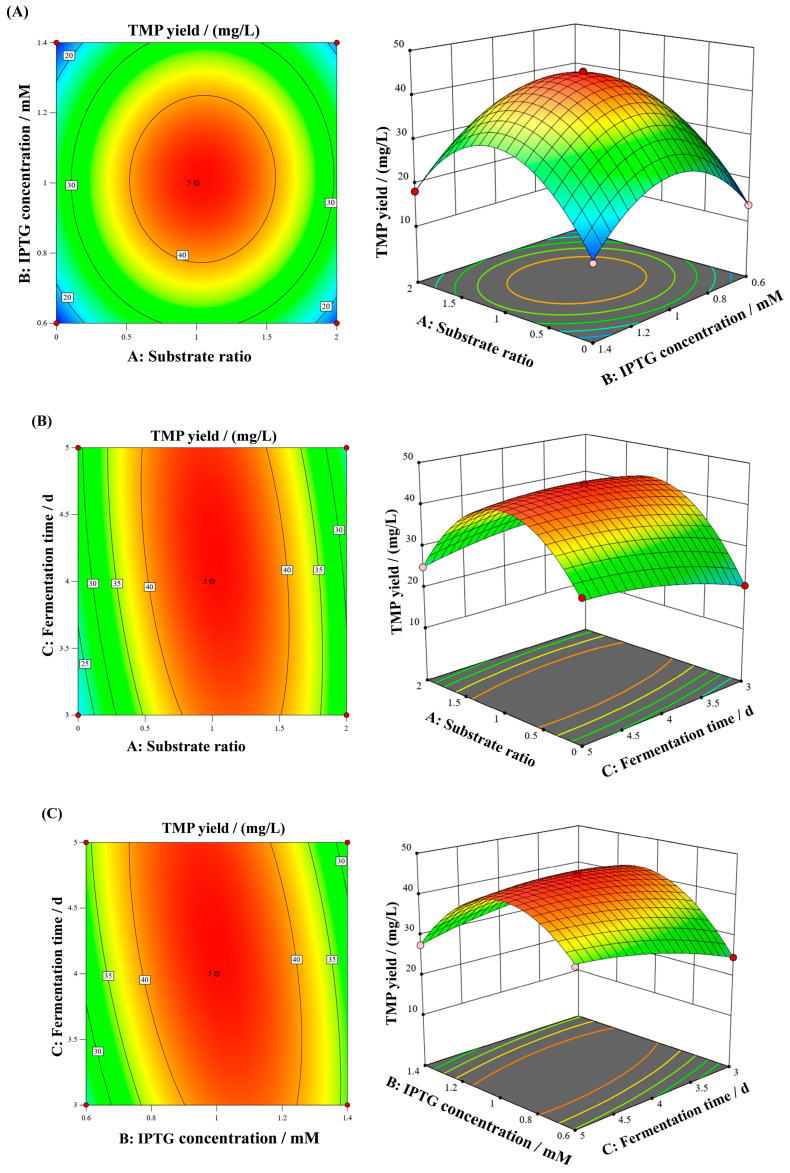
Response surface analysis and contour plots for TMP production. (**A**) Effect of substrate ratio and IPTG concentration on TMP yield; (**B**) effect of substrate ratio and fermentation time on TMP yield; and (**C**) effect of IPTG concentration and fermentation time on TMP yield. The circles in the figure (right side) represent the key points on the response surface, including the optimal point (the top circle) and the extreme points (the three circles at the bottom).

Combining the regression analysis of the response surface and the regression equation, the three-dimensional response surface graph was plotted using Design-Expert 13.0 software. Through the corresponding analysis, the influence of the interaction between the two factors on the TMP yield was explored (Figure 6). When the contour lines of the response surface are circular, it indicates that the interaction between the two factors is weak. When it is oval in shape, it indicates that the interaction is strong and the influence is significant. From Figure 6A, it is shown that the interaction between the substrate ratio and the IPTG concentration was weak. However, the interaction between the substrate ratio and the fermentation time was strong and had a significant impact on the TMP yield (Figure 6B). Similarly, the interaction between the IPTG concentration and the fermentation time also significantly affected the TMP yield (Figure 6C), which was consistent with the results of Table 4. The protruding trend of the response surface graph indicates that the regression model has a maximum response value. The optimal fermentation conditions for TMP production were forecasted by the model: the substrate ratio was 1.021:1.979, the IPTG concentration was 0.997 mM, and the fermentation time was 4.212 d. Under these optimized conditions, the maximum yield of TMP was predicted to be 44.515 mg/L.

### 3.6. Verification Experiments

The reliability of the regression model in the actual situation was verified by validation experiments. Considering the feasibility of the actual operation, the predicted optimal fermentation conditions were slightly adjusted as follows: the substrate ratio was 1:2, the IPTG concentration was 1.0 mM, and the fermentation time was 4 d. Under the conditions, the average yield of TMP was 44.52 ± 0.21 mg/L, which differed from the theoretical value by 0.005 mg/L. The result showed that the established regression model was reliable and the regression equation could be used for the fermentation production of TMP.

## 4. Discussion

TMP is an alkyl pyrazine compound with broad application prospects. It not only plays an important role in food flavor but also exhibits a variety of pharmacological activities. This study aimed to increase the yield of TMP by constructing an engineered strain expressing highly active *BlTDH* (N157A) and optimizing the fermentation conditions. Firstly, the *BlTDH* and *BlTDH* (N157A) genes were cloned into the pHT01 expression vector through molecular cloning technology. Three strains, namely *B. licheniformis* YC7/pHT01, *B. licheniformis* YC7/pHT01-*BlTDH* and *B. licheniformis* YC7/pHT01-*BlTDH* (N157A), were successfully constructed. Subsequently, the TMP yield of these strains was detected by GC-MS. The result of fermentation verification indicated that the overexpression of *BlTDH* (N157A) significantly increased the production of TMP (Figure 4). It was reported that overexpressing *BsTDH* gene effectively enhanced the yield of TMP in *B. subtilis* 168 [19]. In the present study, the TMP yield of the overexpressed strains was significantly higher than that of the control strain (2.35 ± 0.58 mg/L), especially the strain with *BlTDH* (N157A) (15.35 ± 1.51 mg/L). Similarly, Xu et al. [27] successfully improved the synthetic efficiency of 2,5-dimethylpyrazine by screening highly active TDH variants. These results indicated that the *TDH* gene plays a key catalytic role in the alkylpyrazine biosynthesis pathway.

Zhang et al. [19] determined that microorganisms could simultaneously utilize L-threonine and D-glucose or L-threonine alone to generate TMP. Therefore, studying the addition ratio of D-glucose to L-threonine is crucial for optimizing the synthesis of TMP. The single-factor experiments found that with the change in the ratio of D-glucose to L-threonine, the yield of TMP showed a trend of increasing first and then decreasing (Figure 5A). When the substrate ratio was 1:2, the TMP yield was the highest (24.83 ± 0.61 mg/L), indicating that an appropriate and relatively high supply of L-threonine is crucial for increasing TMP production. When only D-glucose was added to the fermentation medium, the strain produced almost no TMP (Figure 5A). The results are highly consistent with the biosynthetic mechanism of TMP proposed by Zhang et al. [19], that is, L-threonine, as the direct substrate of TDH enzyme, plays a central role in the TMP synthesis pathway. D-glucose may mainly support the growth and metabolic activities of bacteria by providing energy and carbon sources [28]. The optimal ratio of 1:2 between glucose and L-threonine reflected the dual metabolic pathways where L-threonine served as the direct substrate for TDH-catalyzed formation of 2-amino-3-ketobutyric acid, while glucose primarily provided energy and carbon skeletons for bacterial growth and cofactor regeneration.

In this study, IPTG, as an inducer, directly affected the expression level of the *TDH* gene and thereby influenced the synthesis efficiency of TMP. The influence of different IPTG concentrations on the yield of TMP was explored through single-factor experiments. When the IPTG concentration was at the intermediate value, 1.0 mM, the TMP yield presented the maximum of 17.43 ± 0.48 mg/L (Figure 5B). This phenomenon might be due to the optimal expression of the *BlTDH* gene at this concentration. However, an excessively high IPTG concentration might cause toxic effects or affect normal physiological functions of cells, ultimately leading to a decrease in TMP production [29]. In *B. subtilis* expressing pHT01-*kIspS*, the optimal induction concentration of IPTG was also 1.0 mM [30]. These results indicated that for the IPTG-induced pHT01 expression system, 1.0 mM IPTG may be the generally applicable optimal concentration, which can provide a reference for the efficient expression of different proteins in *Bacillus* strains.

Fermentation time has an important influence on the accumulation of microbial metabolites and is one of the key parameters for the optimization of fermentation processes [31]. In the study, we also investigated the effect of different fermentation durations on the yield of TMP. It was found that the yield of TMP reached the maximum value (24.70 ± 0.39 mg/L) at 4 d of fermentation (Figure 5C), indicating that the accumulation of TMP requires a certain amount of time. However, as the fermentation progresses, the nutrients in the culture medium are gradually depleted, the cells enter the apoptotic stage, and physiological metabolic activities slow down, thereby affecting the final yield of metabolites [32].

Response surface analysis is a clear, accurate and widely applied method for optimizing fermentation conditions. It has been successfully applied to increase the yield of various enzymes, extracellular polysaccharides, amino acids and other metabolites produced by *Bacillus* [33,34]. In this study, the center point of the response surface design and the levels of each factor were determined through single-factor experiments. Design-Expert 13.0 software was used to draw a three-dimensional response surface diagram to visually present the influence of various factors and their interactions on TMP yield [35]. The results of variance analysis showed that the interaction between substrate ratio and fermentation time, as well as between IPTG concentration and fermentation time, had a significant impact on the yield of TMP (Table 4). These results indicated that the fermentation time not only affected the TMP yield alone, but also interacted with other factors to jointly regulate the synthesis process of TMP. The high R^2^ value (0.9980) and R^2^_Adj_ value (0.9954) indicated that the established regression model had excellent fit and high prediction accuracy, which provided a reliable basis for determining the optimal fermentation conditions. According to the regression model, the optimal conditions of fermentation were predicted and adjusted: the substrate ratio was 1:2, the IPTG was 10 mM, and the fermentation time was 4 d. Ultimately, the verification experiment showed that the yield of TMP was 44.52 ± 0.21 mg/L, which differed from the predicted value by only 0.005 mg/L. The minimal difference between predicted (45.515 mg/L) and actual (44.52 ± 0.21 mg/L) TMP yields demonstrated that such practical adjustments did not significantly compromise optimization benefits. Ultimately, after optimizing the fermentation conditions, the TMP yield increased by 29.17 mg/L compared with the yield (15.35 ± 1.51 mg/L) under the initial condition.

As a food flavor and medicinal compound, TMP has broad application prospects. Recently, researchers have been committed to clarifying the generation mechanism of TMP and developing new strategies to promote its biosynthesis. Through genetic engineering combined with optimization of fermentation conditions, our study confirmed that the highly active TDH (N157A) mutant has great potential to improve TMP synthesis. In the future, the functions and regulatory mechanisms of other key enzymes in the TMP biosynthetic pathway can be further explored. Through multi-gene co-expression or metabolic network reconstruction, more efficient engineered strains can be constructed. Additionally, in the process of large-scale fermentation, the production of TMP may encounter many challenges, such as oxygen mass transfer, substrate inhibition, product feedback inhibition, etc. Researchers should develop corresponding solutions to provide more comprehensive theoretical guidance and technical support for the industrial production of TMP.

## 5. Conclusions

In this study, the *B. licheniformis* YC7 strain expressing BlTDH (N157A) mutant was constructed through molecular cloning. The detection of GC-MS revealed that the TMP yield of the engineered strains containing *BlTDH* (N157A) (15.35 ± 1.51 mg/L) or *BlTDH* (9.86 ± 1.24 mg/L) was significantly higher than that of the control (2.35 ± 0.58 mg/L). It was confirmed that the *TDH* gene plays a key role in the TMP synthesis pathway. To further improve the TMP yield, the effects of substrate ratio, IPTG concentration and fermentation time on the yield of TMP were analyzed by single-factor experiments. Combining the Box–Behnken design and response surface analysis, a reliable regression model was established, with an R^2^ value of 0.9980. Based on the predicted value and the actual situation, the optimal fermentation conditions were finally determined as the substrate ratio of 1:2, the IPTG concentration of 1.0 mM, and the fermentation time of 4 d. Under this combination condition, the yield of TMP reached 44.52 ± 0.21 mg/L. The above results prove the reliability of the regression model and provide a theoretical basis and strain resources for the industrial production of TMP.

## Figures and Tables

**Table 1:** Strains and plasmids used in this study.

Strain/Plasmid	Feature	Source
*B. licheniformis* YC7	TMP-producing bacteria	Laboratory preservation
*E. coli* DH5α	Plasmid cloned bacteria	Laboratory preservation
*B. licheniformis* YC7/pHT01	Strain with blank plasmid	This study
*B. licheniformis* YC7/pHT01-*BlTDH*	Recombinant strain	This study
*B. licheniformis* YC7/pHT01-*BlTDH* (N157A)	Recombinant strain	This study
pHT01	Expression plasmid	This study
pET-28a-*BlTDH*	Recombinant plasmid	Laboratory preservation
pET-28a-*BlTDH* (N157A)	Recombinant plasmid	Laboratory preservation
pHT01-*BlTDH*	Recombinant plasmid	This study
pHT01-*BlTDH* (N157A)	Recombinant plasmid	This study

**Table 2:** The selected levels of three factors in the Box–Behnken design.

Factor	Level		
	−1	0	1
(A) Substrate ratio	0:1	1:2	2:1
(B) IPTG concentration (mM)	0.6	1.0	1.4
(C) Fermentation time (d)	3	4	5

**Table 3:** Box–Behnken design and determination of TMP yield.

Test Number	Independent Variables			TMP Yield (mg/L)
	A: Substrate Ratio	B: IPTG Concenteation (mM)	C: Fermentation Time (d)	
1	0:1	0.6	4	13.84
2	2:1	0.6	4	16.05
3	0:1	1.4	4	13.52
4	2:1	1.4	4	18.14
5	0:1	1.0	3	20.68
6	2:1	1.0	3	29.55
7	0:1	1.0	5	29.59
8	2:1	1.0	5	24.96
9	1:2	0.6	3	24.74
10	1:2	1.4	3	34.46
11	1:2	0.6	5	33.63
12	1:2	1.4	5	27.56
13	1:2	1.0	4	44.76
14	1:2	1.0	4	44.57
15	1:2	1.0	4	44.99
16	1:2	1.0	4	44.67
17	1:2	1.0	4	43.11

**Table 4:** Analysis of variance of the response surface regression model.

Source	Sum of Squares	Degrees of Freedom	Mean Square	F-Value	*p*-Value	Significance
Model	2084.96	9	231.66	386.06	<0.0001	**
A	15.32	1	15.32	25.53	0.0015	**
B	3.67	1	3.67	6.12	0.0426	*
C	4.98	1	4.98	8.29	0.0237	*
AB	1.45	1	1.45	2.42	0.1638	
AC	45.56	1	45.56	75.93	<0.0001	**
BC	62.33	1	62.33	103.87	<0.0001	**
A2	1141.80	1	1141.80	1902.77	<0.0001	**
B2	664.75	1	664.75	1107.79	<0.0001	**
C2	13.01	1	13.01	21.67	0.0023	**
Residual	4.2	7	0.6001			
Lack of fit	1.96	3	0.6530	1.17	0.4266	
Pure error	2.24	4	0.5604			
Cor total	2089.16	16				
R^2^	0.9980					
R^2^_Adj_	0.9954					
R^2^_pred_	0.9833					

“**” indicates an extremely significant difference between the results (*p* < 0.01), and “*” indicates a significant difference between the results (*p* < 0.05).

## Data Availability

The original contributions presented in this study are included in the article. Further inquiries can be directed to the corresponding author.
